# The Composition and Anti-Aging Activities of Polyphenol Extract from *Phyllanthus emblica* L. Fruit

**DOI:** 10.3390/nu14040857

**Published:** 2022-02-18

**Authors:** Min Wu, Jianhang Cai, Zhengfeng Fang, Shanshan Li, Zhiqing Huang, Zizhong Tang, Qingying Luo, Hong Chen

**Affiliations:** 1 College of Food Science, Sichuan Agricultural University, Ya’an 625014, China; wumin@stu.sicau.edu.cn (M.W.); 202005149@stu.sicau.edu.cn (J.C.); zfang@sicau.edu.cn (Z.F.); b20172202@stu.sicau.edu.cn (S.L.); 14186@sicau.edu.cn (Q.L.); 2 Institute of Animal Nutrition, Sichuan Agricultural University, Chengdu 611134, China; zqhuang@sicau.edu.cn; 3 College of Life Sciences, Sichuan Agricultural University, Ya’an 625014, China; 14126@sicau.edu.cn

**Keywords:** medicine and food homologous plant substances, natural products, bioactivity evaluation, polyphenols, antioxidant, anti-aging

## Abstract

*Phyllanthus emblica* L. (PE) is commonly known as a medicine and food homologous plant, which is abundant in natural products polyphenols. In the present study, polyphenols were extracted from PE fruit by response surface method, and the anti-aging ability was determined. PE fruit polyphenols exhibited strong antioxidant capacities in scavenging free radicals, and anti-cholinesterase ability by inhibition of AChE (IC_50_ 0.2186 ± 0.0416 mg/mL) and BuChE (IC_50_ 0.0542 ± 0.0054 mg/mL) in vitro. Moreover, PE fruit polyphenols showed strong protective effect against the aging process in *Caenorhabditis elegans* model, including increased thermal resistance, extended lifespan by 18.53% (*p* < 0.05), reduced activity of AChE by 34.71% and BuChE by 45.38% (*p* < 0.01). This was accompanied by the enhancement in antioxidant enzymes activity of SOD by 30.74% (*p* < 0.05) and CAT by 8.42% (*p* > 0.05), while decrease in MDA level by 36.25% (*p* < 0.05). These properties might be interrelated with the presence of abundant flavonols and phenolic acids identified by UPLC-ESI-QTOF-MS, such as quercetin, myricetin, ellagic, gallic, and chlorogenic acids, together with their glycosides. The remarkable antioxidant and anti-aging potential of PE fruit polyphenols could be implemented in the food and pharmaceutical industry.

## 1. Introduction

Aging is an inevitable biological process that affects the health of an increasing number of aged individuals worldwide, characterized by the progressive loss of structural, functional, and physiological integrity [1,2]. The main determinant of aging is recognized to be the constant oxidative damage, due to the accumulation of excessive free radicals in cellular components [3]. Especially, the brain contains abnormally high proportions of polyunsaturated fatty acids, making this organ a common target for oxidative damage response. Sustained damage results in deficit of choline and neuronal death, thus leading to the occurrence and development of aging and age-related neurodegenerative diseases, such as Alzheimer’s disease (AD) [4]. During this process, enzymes acetylcholinesterase (AChE) and butyrylcholinesterase (BuChE) play an important role in the hydrolysis of choline and accelerating aging [5]. In response to stress, the organisms correspondingly activate intercellular antioxidant enzyme systems, such as superoxide dismutase (SOD) and catalase (CAT), glutathione peroxidase. These defense systems are accordingly employed in blocking the damage response, and timely repair of damaged cells to maintaining the oxidative equilibrium and preventing aging [6]. However, when the endogenous antioxidant system is limited with increased age, supplementation of exogenous antioxidants to evoke antioxidant systems seems to be a focus to defer aging and promote health.

Recently, natural products polyphenols have received increasing interest as strong exogenous antioxidants. They have been reported to possess antioxidant, anti-inflammatory, anti-microbial, anti-cancer, anti-diabetic properties, etc. These properties are implicated in the reduced risk of aging process and various age-related diseases [2,7]. Polyphenols are abundant in *Phyllanthus emblica* (PE), a plant belonging to the *Phyllanthaceae* family. PE is widely distributed in subtropical and tropical areas, such as China, India, and the Malay Peninsula [8]. For centuries, PE fruit is used not only as food but also as traditional Chinese medicine, wherein it is regarded as “the best among rejuvenators” [9]. The chemical components of PE fruit were abundant in polyphenols, vitamin C, minerals, and other metabolites, such as lactones, terpenoids, and alkaloid [10]. Among these, polyphenols were thought to be the crucial bioactive compounds underlying the health-promoting effects of PE fruit.

The purpose of the present study was to identify the active components in polyphenols extracted from PE fruit and evaluate its anti-aging activities. *Caenorhabditis elegans* (*C. elegans*) was used as a model to investigate the anti-aging potential and the related mechanisms of PE fruit polyphenols in vivo. This work is the first to systematically evaluate the antioxidant and anti-aging activities of polyphenols isolated from PE fruit both in vitro and in vivo. Our data have confirmed the potential of PE fruit polyphenols to decelerate aging process and benefit human health, suggesting that PE fruit polyphenols could be implemented in the food and pharmaceutical industry.

## 2. Materials and Methods

### 2.1. Chemicals and Reagents

Folin–Ciocalteu reagent, salicylic acid, Na_2_CO_3_, ascorbic acid (Vc), ethanol, H_2_O_2_ solution (30%, *w*/*w*), and other reagents (analytical grade) were acquired from Sinopharm Chemical Reagent Co., Ltd. (Chengdu, China). Gallic acid, DPPH, ABTS, 2,4,6-Tris(2-pyridyl)-S-triazine (TPTZ), ACHE from electric eel, BuChE from equine serum, and donepezil hydrochloride were obtained from Yuan Ye Biological Technology Company Ltd. In Shanghai, China.

### 2.2. Preparation of PE Fruit Polyphenol

Fresh PE fruits were collected from Shantou City, Guangdong Province, China. Then were cleaned, pitted, and dried at 45 °C for 36 h. The dry pulp was then ground into powder and passed through a 100-mesh sieve for subsequent experiments. The powder was dissolved in ethanol–water mixture (45%, *v*/*v*) and stirred on a rotary shaker (120 rpm) to extract polyphenols. The residue was re-extracted and then filtered with a filter paper. The filtrates were combined and concentrated using a rotary evaporator at 45 °C. The concentrated extract was precipitated by adding three-fold volume of absolute alcohol. The precipitants were then centrifuged at 10,000 rpm for 10 min at 4 °C, and the resulted supernatant was freeze-dried at −80 °C in vacuum.

### 2.3. Determination of Total Polyphenol Content (TPC)

Folin–Ciocalteu reagent (500 μL) was added into gallic acid solutions, mixed thoroughly for 5 min, and first 4.5 mL of 7.5% Na_2_CO_3_ and then distilled water were added, and incubated at room temperature for 60 min. Absorbance was measured at 740 nm on a UV-visible spectrophotometer (UV-1780, Shimadzu, Japan). TPC was expressed as mg of gallic acid equivalents (GAE)/g of powder on dry weight (DW). The obtained standard curve equation was shown as Equation (1):

(1) $$y=0.1721x+{0.0085\;(R}^{2}=0.9994)$$

### 2.4. Optimization of Polyphenol Extraction from PE Fruit

#### 2.4.1. Single Factor Extraction Experiments

In brief, 1 g of PE fruit powder was dissolved in 25 mL of 45% ethanol and stirred under 40 °C for 135 min to extract polyphenols. Different extraction times (45, 90, 135, 180, and 225 min), extraction temperatures (30 °C, 40 °C, 50 °C, 60 °C, and 70 °C), ethanol concentrations (15%, 30%, 45%, 60%, and 75%, *v*/*v*), and liquid–solid ratios (15, 20, 25, 30, and 35 mL/g) were evaluated individually to determine the optimum extraction conditions. The parameters that influenced the most on TPC yield were selected for subsequent response surface method (RSM) experiments.

#### 2.4.2. Experimental Design for RSM

On the basis of the single-factor extraction experiments, a Box–Behnken design (BBD) with independent three variables and 17 runs was used to determine the response pattern using Design-Expert 8.0.6 (Stat-Ease Inc., Minneapolis, MN, USA). The response variables were fitted to a second-order polynomial model equation obtained by RSM shown as Equation (2):

(2) $${Y=\beta }_{0}+\overset{2}{\underset{i=1}{\sum }}\beta_{i}X_{i}+\overset{2}{\underset{i=1}{\sum }}\beta_{\mathrm{ii}}X_{i}^{2}+\underset{i}{\sum }\underset{j=i+1}{\sum }\beta_{\mathrm{ij}}X_{i}X_{j}$$

Response variables (Y) were extraction yield of TPC, X_i_ and X_j_ were the independent factors affecting the response. β_0_, β_i_, β_ii_, and β_ij_ were the regression coefficients of the model (intercept, linear, quadratic, and interaction term).

### 2.5. Identification of Phenolic Compounds by UPLC-ESI-QTOF-MS

PE fruit polyphenols were dissolved in 80% methanol and ultrasonically treated at 4 °C for 30 min. The mixture was then vortexed for 30 s and centrifuged at 12,000 rpm for 15 min at 4 °C. In brief, 5 μL of internal standard (140 μg/m DL-o-chlorophenylalanine) was added to 200 μL of polyphenol supernatant for analysis on LC-ESI/MS (Waters ACQUITY UPLC-XEVO G2-XS QTOF) equipped with a ACQUITY UPLC BEH C_18_ column (1.7 µm, 2.1 × 100 mm). Chromatographic operating conditions are as follows: column temperature: 45 °C; flow rate: 0.4 mL min^−1^; mobile phase A: water + 0.1% formic acid; mobile phase B: acetonitrile + 0.1% formic acid; and injection volume: 0.5 μL. Gradient was set as follows: 0–0.5 min: 99% A and 1% B; 0.5–2.5 min: 90% A and 10% B; 2.5–7 min: 75% A and 25% B; 7–10.5 min: 15% A and 85% B; 10.5–14.5 min: 100% B; and 14.5–16 min: 99% A and 1% B. Nitrogen was used as drying, nebulizer, and collision gas. The collision energy was 6 eV (low) and 20–40 eV (high). The spectra were calibrated by 250 pg/uL leucine enkephalin. MS was operated in negative and positive electrospray ionization modes, and spectra were recorded by scanning the mass range from m/z 50 to 1500. Data were converted to ABF using ABFConverter.4.0.0 (Thermo, Waltham, MA, USA). Feature data were extracted, pre-processed, normalized, and processed into 2D data matrix using Excel 2010 software. The phenolic compounds were identified by comparing the retention times, peak spiking, and mass spectra with those found in literature and databases, such as SciFinder-Scholar (https://scifinder.-cas.org (accessed on 14 June 2021)), Phenol-Explorer (www.phenol-explorer.eu (accessed on 25 October 2021)), MassBank (http://www.massbank.jp/ (accessed on 25 June 2021)), Human Metabolome Database (https://hmdb.ca/ (accessed on 14 June 2021)), and PubChem (https://pubchem.ncbi.nlm.nih.gov (accessed on 25 December 2021)) [11].

### 2.6. In Vitro Antioxidant Assays

#### 2.6.1. DPPH Radical Scavenging Assay

In brief, 200 μL of ethanol was added to 200 μL of 0.2 mM DPPH ethanol solution and 600 μL of PE fruit polyphenols (0–0.15 mg/mL). After incubation in the dark for 30 min, the absorbance was recorded at 517 nm with the automatic microplate reader (Thermo Fisher Scientific Inc., Waltham, MA, USA). Vc was used as a positive control. DPPH radical scavenging ability was calculated and expressed as the following Equation (3):

(3) $$\mathrm{Scavenged}\;\mathrm{rate}\;(\%)=\frac{A_{0}\;-\;(A_{S}\;-\;A_{1})}{A_{0}}\;\times \;100\%$$

where A_0_ is the absorbance of the control reaction (containing all reagents except the test compound), A_1_ is the absorbance of the background reaction (containing all reagents except DPPH ethanol solution), and A_S_ is the absorbance with the test compound.

#### 2.6.2. ABTS·^+^ Radical Scavenging Assay

ABTS·^+^ solution was generated by mixing and reacting potassium persulfate (2.45 mM) and ABTS·^+^ (7 mM) in a ratio of 1:1. The mixture was kept in the dark for 12 h before use. Diluted ABTS·^+^ solutions were prepared in ethanol until a value of 0.700 ± 0.002 was reached at 732 nm absorbance. Afterward, 40 μL of PE fruit polyphenols (0–0.15 mg/mL) was added to 300 μL of ABTS·^+^ solution. After incubation in the dark for 30 min, the absorbance was recorded at 732 nm. ABTS·^+^ radical scavenging ability was calculated and expressed as Equation (3).

#### 2.6.3. OH· Radical Scavenging Assay

In brief, 200 μL of PE fruit polyphenols (0–1.2 mg/mL), 200 μL of FeSO_4_ (9 mM), and 200 μL of H_2_O_2_ (8.8 mM) were mixed. A reaction was initiated by the addition of 200 μL of salicylic acid (9 mM) and water. Following the mixture and incubation for 30 min at 37 °C, the absorbance was measured at 510 nm. OH· radical scavenging ability was calculated and expressed as Equation (3).

#### 2.6.4. FRAP Assay

Working FRAP reagent was prepared daily by mixing acetate buffer (300 mM, pH 3.6), TPTZ solution (10 mM in 40 mM HCl), and FeCl_3_·6H_2_O solution (20 mM) in 10:1:1 *v*/*v* ratio. The mixture was warmed at 37 °C. Afterward, 50 µL of PE fruit polyphenols (0–0.15 mg/mL) was added to 1.5 mL of FRAP reagent, and the absorbance was measured after incubation for 30 min at 37 °C at 593 nm. FRAP rate was calculated and expressed as Equation (3).

### 2.7. Inhibition of Cholinesterase Activity Assay In Vitro

AChE/BuChE inhibitory assay was performed according to Ellman method with slight modifications [12]. Donepezil hydrochloride was used as a positive control. In brief, 60 µL of 0.1 M PBS (pH 7.4) was mixed with 60 µL of test sample (0–1.2 mg/mL PE fruit polyphenols), 300 µL of DTNB (1 mM), and 120 µL of AChE/BuChE in Tris-HCl buffer (0.02 M, pH 7.5). After 15 min of incubation in the dark at 37 °C, the reaction was initiated by the addition of 60 µL of ATCI/BTCI (1 mM). Absorbances were measured at 405 nm, followed by another incubation in the dark for 8 min at 37 °C. AChE/BuChE inhibition rate was calculated using Equation (4):

(4) $$\mathrm{Inhibition}\;\mathrm{rate}\;(\%)=\left(1\;-\;\frac{A_{S}\;-\;A_{1}}{A_{0}\;-\;A_{0^{\prime }}}\right)\;\times \;100\%\;$$

where A_S_ is absorbance with sample, A_1_ is absorbance with sample and without AChE/BuChE, A_0_ is absorbance without sample, and A_0′_ is absorbance without sample and AChE/BuChE.

### 2.8. In Vivo Assays

#### 2.8.1. *C. elegans* Strains and Maintenance

Wild-type *C. elegans* (N2) strain was procured from Caenorhabditis Genetics Center (University of Minnesota, USA). Strains were grown and maintained on nematode growth medium (NGM) agar plates seeded with *E. coli* OP_50_ at 20 °C.

#### 2.8.2. Thermal Stress Resistance Assay

The age-synchronized *C. elegans* were incubated on NGM plates containing heat-killed *E. coli* OP50 and different concentrations of PE fruit polyphenols (0, 0.1, 0.2, 0.4, 0.8 and 1.2 mg/mL, diluted in DMSO to obtain a final concentration of 0.1% DMSO) till they reached L4 stage. The plates having 0.1% DMSO (*v*/*v*) were kept as control plates. Then, the pretreated synchronization L4 worms (*n* = 30) were transferred to 35 °C for 6 h, and the dead was counted every 2 h. The worms were scored as dead when they failed to respond to repeated touch with platinum loop.

#### 2.8.3. Lifespan Assay

The lifespan assays were performed using 50 synchronized L4 *C. elegans*. They were transferred to NGM plate with PE fruit polyphenols and incubated at 20 °C. *C. elegans* were transferred to new plates every 1–2 days, depending on prevalent mortality and surviving nematodes were counted daily until all nematodes were dead. The percentage of live *C. elegans* at the given time points are represented by the depicted survival curves.

#### 2.8.4. Determination of Cholinesterase Activities

Approximately, 2500 synchronized *C. elegans* were treated as described above. On reaching the L4 stage, they were collected by washing with M9 buffer and subsequently transferred to microtubes. Next, the worms were homogenized on ice to break the cuticle. The mixture was centrifuged at 2500 r/min for 10 min at 4 °C and upper aqueous layer was transferred to a new microtube for enzymatic assay [13]. The activities of AChE and BuChE, and protein content were measured using commercial chemical assay kits (Nanjing Jiancheng Bioengineering Institute, Nanjing, China).

#### 2.8.5. Determination of Antioxidant Enzyme Activities and MDA Levels

Experimental conditions were described according to “2.8.4”. The SOD and CAT activities, and malondialdehyde (MDA) content were determined using commercial kits.

### 2.9. Statistical Analysis

The survival analyses were performed using the Kaplan–Meier method by GraphPad Prism 7 software (San Diego, CA, USA) and the statistical analyses were analyzed using IBM SPSS 19 (Armonk, NY, USA). Statistical significance was determined by one-way analysis of variance (ANOVA) with Duncan’s multiple comparison post-test and differences were considered to be significant at *p* < 0.05.

## 3. Results and Discussion

### 3.1. Optimizing the Extraction of PE Fruit Polyphenols

**Figure 1:**
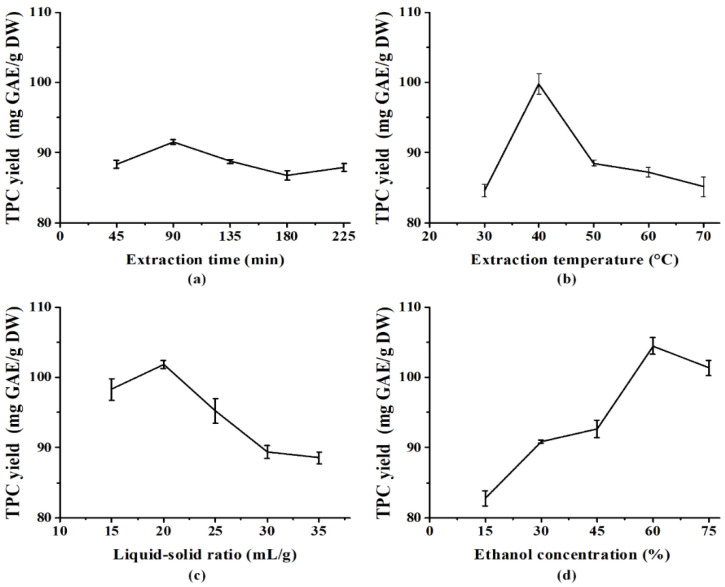
Single-factor experiment on the effect of extraction time (**a**), ethanol concentration (**b**), liquid–solid ratio (**c**), and extraction temperature (**d**) on the extraction yield of total phenolics from PE fruit (*n* = 3).

Herein, oscillation-assisted solid–liquid method was adopted to extract the polyphenols from PE fruit by ethanol. Single-factor experimental results showed that the extraction time had the least influence on TPC yield (Figure 1) and hence was maintained at 90 min for the following RSM experiments. Table 1 shows the experimental results of BBD with three independent variables and 17 runs fitted to a second-order polynomial model shown as Equation (5):

(5) $$Y=92.27\;-\;6.78A+5.04B\;-\;0.19C\;-\;5.23\mathrm{AB}+0.0025\mathrm{AC}\;-\;0.09\mathrm{BC}\;-\;{0.2A}^{2}+{3.89B}^{2}+{1.83C}^{2}$$

where Y was the extraction yield of TPC (mg GAE/g DW), A, B, and C were the coded values of the tested ethanol concentration (%), liquid–solid ratio (mL/g), and extraction temperature (°C), respectively.

**Figure 2:**
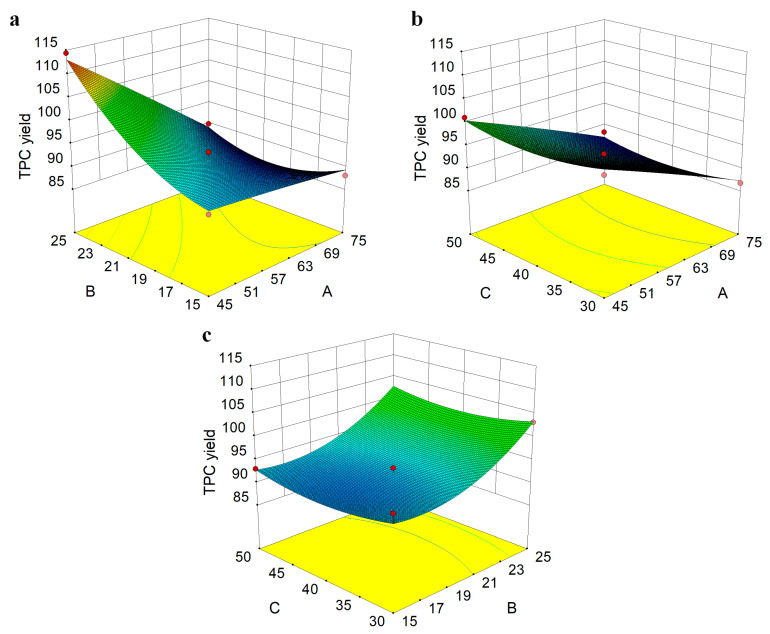
The three-dimensional response surface methodology of various factors on TPC in PE fruit, ethanol concentration and liquid-solid ratio (**a**), ethanol concentration and extraction temperature (**b**), liquid-solid ratio and extraction temperature (**c**).

**Figure 3:**
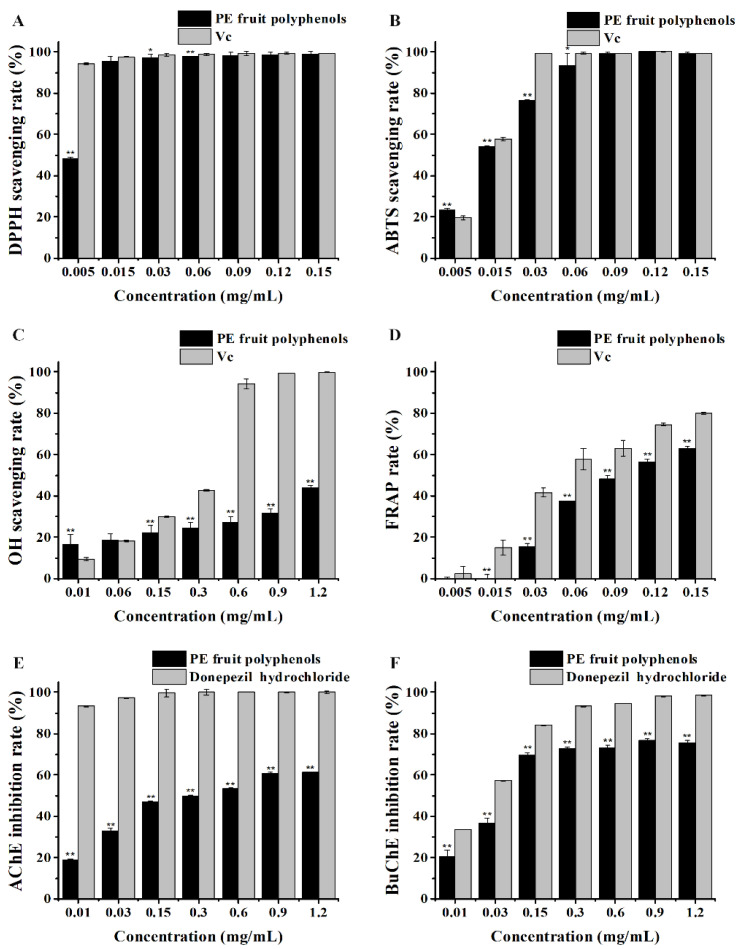
Antioxidant and anti-aging activity of polyphenols from PE fruit ((**A**): DPPH free radical scavenging activity; (**B**): ABTS·^+^ free radical scavenging activity; (**C**): OH· free radical scavenging activity; (**D**): ferric ion reducing power; (**E**): AChE inhibition activity; (**F**): BuChE inhibition activity). * *p* < 0.05 was significant from the control, and ** *p* < 0.01 was extremely significant from the control.

It was observed that the regression variance model of *p* < 0.01, indicating that our model was extremely significant (Table S1). Surface variance analysis revealed that the lack of fit of the equation was not significant (*p* > 0.05), and the correction coefficient squared R^2^ = 0.9747, R^2^_Adj_ = 0.9423, indicating that this equation was well fitted to the test. The descending order of the factors affecting the TPC yield was ethanol concentration > liquid–solid ratio > extraction temperature. According to the regression equation, the shape of the fitted response surface was investigated, and the interaction of various factors on the TPC in PE fruit was analyzed. As shown in Figure 2, the influence of each variable on TPC showed initially a trend of increase and then decrease, which may be due to the decomposition of phenolic compounds. The steeper the slope of the response surface, the more significant the interaction [14]. With the change of ethanol concentration (A) and liquid–solid ratio (B), the response surface tended to be parabola, and the slope of the surface was steep, indicating that there was an interaction between A and B in Figure 3A, which was consistent with those of ANOVA (P_AB_ < 0.01). In order to validate the prediction accuracy of the mathematical model, validation experiments were conducted under the optimum extraction conditions predicted by RSM with slight modifications: ethanol concentration of 45%, liquid–solid ratio of 25 mL/g, and extraction temperature of 50 °C. Under these conditions, the actual TPC extraction yield was 114.01 ± 2.31 mg GAE/g DW (*n* = 5), which highly corresponded with the predicted yield (114.35 mg GAE/g DW) by the regression model. This TPC value was higher than the 42.78 and 72.91 mg GAE/g DW obtained by Sousa, et al. and Syed Mubashar Sabir, et al., respectively [15,16]. These results indicated that the model could accurately and adequately predict the extraction conditions of polyphenol from PE fruit.

### 3.2. Phenolic Compounds of PE

In this work, the PE fruit polyphenol composition was subjected to ultra-high-performance liquid chromatography coupled with quadrupole time-of-flight mass spectrometry (UPLC-ESI-QTOF-MS). There were approximately 300 mass spectrum outputs studied for each analytical replicate, allowing for the tentative identification of up to 84 phenolic compounds. Table 2 lists the identified phenolic compounds together with their corresponding Rt, observed (*m*/*z*) [M-H]^−/+^, and molecular formula. One of the most abundant groups in the phenolics from PE fruit was phenolic acids, including 15 types of hydroxybenzoic acids and 10 types of hydroxycinnamic acids. Some of the hydroxybenzoic acids were gallic acid (2), a marker phenolic compound of PE, and its derivates such as 3-O-methylgallate (3), syringic acid (4), 4-O-methylgallic acid (6), ellagic acid (7), beta-glucogallin (8), and chebulic acid (15). These identified hydroxybenzoic acids are common phenolics in PE fruit [17,18,19]. Some of the identified hydroxycinnamic acids were caffeic acid (17) and its derivatives caftaric acid (18), 2-O-caffeoylhydroxycitric acid (19), caffeic acid 3-O-glucuronide (22)**,** and chlorogenic acid (24). These compounds are present in many plants, but only caffeic acid and chlorogenic acid are previously detected in PE by [17,20].

Flavonols (38–66) are one of the most abundant groups of flavonoids found in PE, and the majority of which are present in the form of glycosides. Eight of the compounds were determined as quercetin (42) and its derivatives (41, 44, 46, 52, 61–62, and 66), seven as myricetin (47) and its derivatives (54, 58, 60, and 63–65), and three as kaempferol derivatives (43, 53, and 59). These compounds are typical flavonols found in PE [20,21]. However, other flavonols, such as limocitrol (38), fisetin (39), afzelin (40)**,** and spiraeoside (45) have not been detected in PE fruit until now. In addition, eight flavanones were identified as naringenin (26), naringenin-7-O-glucoside (28), and hesperetin glycosides (32–33) previously detected in other edible plants [11,22]. Flavan-3-ols were identified as simple monomers (-)-epigallocatechin 3′-glucuronide (34), leucodelphidin (35), theaflavin 3,3′-digallate (36), and oligomeric tannin (37). These results showed that the positive mode analysis was appropriate for anthocyanins, dihydroflavonols, and flavones due to their behavior in the liquid phase [16]. Anthocyanins including cyanidin, pelargonidin, delphinidin, and peonidin (67–72), were found to bond to various glycosides. Several dihyflavonols, flavones, and isoflavones were tentatively identified in PE, such as neoastilbin (73), scutellarein 5-glucuronide (74), 2″-O-acetylisoorientin (75), daidzein 4′-O-glucuronide (79), ononin (80), medicarpin 3-O-(6′-malonylglucoside) (81), and Osajin (82). In general, these results provided a database for structural analysis of active phenolic compounds responsible for beneficial health effects.

### 3.3. In Vitro Antioxidant Activity of PE Fruit Polyphenols

The elevated levels of free radicals are the major cause of cellular oxidative damages and further implicated in human biological aging. Here, different antioxidant methods in vitro including DPPH, ABTS·^+^, OH· radical scavenging, and FRAP assays were performed to analyze the antioxidant activities of PE fruit polyphenols. According to DPPH radical scavenging assay, a dose-dependent increase in scavenging rate by polyphenols was recorded from 0.005 mg/mL to 0.015 mg/mL. At concentrations higher than 0.015 mg/mL, the scavenging rate of PE fruit polyphenols and Vc was both higher than 90% (Figure 3A). The IC_50_ value obtained for PE fruit polyphenols was 0.0033 ± 0.0003 mg/mL, whereas that for Vc was 0.0003 ± 0.0001 mg/mL. A similar trend for the ABTS·^+^ radical scavenging was observed (Figure 3B). As illustrated in Figure 3C, the OH· radial scavenging rate of PE fruit polyphenols and Vc dose-dependently increased (0.01 mg/mL to 0.6 mg/mL), but the former had comparatively lower scavenging rates than the latter at the same concentration. As a result, PE fruit polyphenols had higher IC_50_ value (1.2645 ± 0.0857 mg/mL) for OH· radical than Vc (0.2158 ± 0.0082 mg/mL). A high percentage indicates a high reducing power for antioxidants. Similar to the results from OH· radical scavenging assay, the FRAP percentage of polyphenols was lower than that of Vc (Figure 3D). These results confirmed that PE fruit polyphenols extract had strong antioxidant capacity in vitro, among that the scavenging ability against DPPH and ABTS·^+^ radicals was almost comparable with that of Vc.

Polyphenols represent a broad group of heterogeneous compounds marked by hydroxylated phenyl. Their structure contains a number of hydroxyl groups that can provide hydrogen ions and then form strong coordination oxygen ion complexes with metal ion. Consequently, these polyphenols can directly trap free radicals and/or act through metal chelation, leading to the termination of free radical chain reactions [2,23]. In this study, flavanols and phenolic acids were identified as the most abundant phenolic compounds in PE fruits. Among the flavonols, the compounds quercetin, myricetin, fisetin, and nelumboside, were revealed to possess strong antioxidant potential [24,25]. The preferred site of flavanols to inhibit oxidative stress was reported to be the 3-hydroxyl and 4-carbonyl group [25]. Moreover, flavonoid-glycosides have many active groups, such as O-dihydroxyl groups in the B-ring, 2, 3-double bond in the C-ring, and hydroxyl groups at positions 3 in the C-ring and 5 in the A-ring; these are important features for antioxidant activity [26]. The predominant phenolic acids are also responsible for the antioxidant property due to a large number of free hydroxyl groups bonded to their aromatic ring, such as gallic acid, together with its derivatives beta-glucogallin, 2-O-galloylgalactaric acid, 1-methyl 2-galloylgalactarate, and 1-O,6-O-digalloyl-beta-D-glucose [27]. In addition, the ortho-hydroxyl structures leading to the formation of ortho-quinone can increase the antioxidant activity of phenolic compounds [21]. Therefore, the remarkable antioxidant activity of PE fruit polyphenols can also be attributed to the presence of ellagic acid, chlorogenic acid and chebulic acid.

### 3.4. In Vitro Anti-Aging Activity of PE Fruit Polyphenols

Anti-aging activity was analyzed as the ability to inhibit cholinesterase AChE and BuChE, and the results as inhibition rate (%) are presented in Figure 3E,F. It was shown that there was a concentration-dependent increase in inhibition rate against cholinesterase by PE fruit polyphenols from 0 to 0.15 mg/mL. At a concentration of 1.2 mg/mL PE fruit polyphenols, the activity of AChE reduced by (61.23 ± 0.0014)%, while the BuChE inhibition ratio was (75.76 ± 0.0107)%. As a result, the calculated IC_50_ value for AChE was 0.2186 ± 0.0416 mg/mL, whereas that for BuChE was 0.0542 ± 0.0054 mg/mL. The results showed that PE fruit polyphenols had a more pronounced inhibition ability of BuChE than that of AChE, which was in line with the research of Tkacz et al. [28].

Despite the fact that AChE and BuChE have many structural similarities, the inhibitory effects of phenolic compounds are associated with the structure of the enzymes, e.g., active site and oxyanion hole. Our data showed PE fruit polyphenols had different effects on AChE and BuChE, may be owing to that BuChE has a wider range of acyl binding sites than AChE, thus leading to BuChE being more easily recognized and bound with substrates [28]. Phenolic compounds interact with the amino acid residues of AChE and BuChE through the formation of hydrogen bond, π-π, and hydrophobic interactions. The methoxy and hydroxyl groups in polyphenols enhance the enzyme inhibitory effect due to strong binding capacity [28]. Thus, the presence of predominant flavonols, phenolic acids, and anthocyanidins (such as p-coumaric acid, quercetin, kaempferol, myricetin, delphinidin, pelargonidin, cyanidins) might explain the remarkable inhibition activity of PE fruit polyphenols against AChE and BuChE [29]. Our finding stated that PE fruit polyphenol extract could conduct as a source of anti-cholinesterase inhibitors with the potential to delay aging process and improve health.

### 3.5. In Vivo Biological Activity Analysis

#### 3.5.1. PE Fruit Polyphenols Increased Thermal Resistance in *C. elegans*

**Figure 4:**
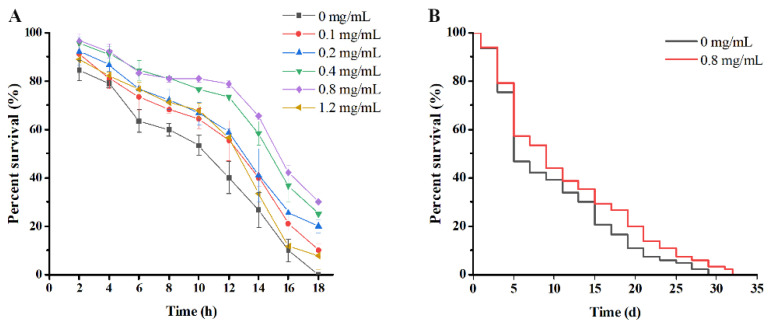
Effect of PE fruit polyphenols on the thermal resistance (**A**) and lifespan (**B**) in *C. elegans*.

*C. elegans*, a member of the phylum *Nematoda*, has the advantages of easy cultivation, observation, short life cycle, and fast reproduction. There is 83% homology in the proteome of nematodes and human genes, thus sharing many similarities in morphological and functional senescence [6,30]. In light of the strong anti-oxidant and anti-cholinesterase effects of PE fruit polyphenols observed in vitro, we extended our studies to study the effects on age-related aspects by using *C. elegans* as a model. Thermotolerance is an important indicator used to understand the aging process. The improved heat-stress resistance helps the nematodes to cope with rough situation to enhance their vitality [31]. As shown in Figure 4A, all test doses of PE fruit polyphenols contributed to a rise of the survival rate in *C. elegans* versus control. Under the heat stress, when all control worms died after 18 h, the mean survival rates of treated group were approximately 10% (0.1 mg/mL), 20% (0.2 mg/mL), 25% (0.4 mg/mL), and 30% (0.8 mg/mL), respectively. However, when higher than 0.8 mg/mL, the mean survival rates in 1.2 mg/mL treated worms were dramatically decreased to 7.8%. This might be due to the higher extracellular osmotic pressure caused by the high concentration polyphenols, thereby affecting the normal physiological activities of worms [32]. The results suggested that 0.8 mg/mL PE fruit polyphenols was a high-efficiency concentration for *C. elegans* to survive, which was selected for the following tests.

#### 3.5.2. PE Fruit Polyphenols Prolonged Lifespan of *C. elegans*

Lifespan represents the most intuitive evaluation index in the process of aging in nematodes. Herein, the lifespan assay was conducted using the optimum dose of PE fruit polyphenols (0.8 mg/mL) to feed *C. elegans*. As shown in Figure 4B, the survival curve of treated group shifted to the right, indicating that the PE fruit polyphenols prominently prolonged the lifespan in worms. The mean lifespan of treated group was increased significantly by 18.53%, and the median lifespan was prolongated up to 16.67% in comparison to the control group (*p* < 0.05). Our data showed that PE fruit polyphenols could improve thermotolerance and extend the lifespan of *C. elegans*, which were the marks of decelerated aging.

Numerous studies revealed that the longevity effect of polyphenols mainly depended on their molecular structure. Grünz et al. [33] reported that the lifespan of worms relied on the hydroxyl-group of the C-ring in the flavonoids, and further increased with the number of hydroxyl-groups attached to the B-ring. Hence myricetin showed the strongest effect on the lifespan-extending followed by quercetin, kaempferol, and naringenin. Additionally, the glycosylation of the flavonols made a difference to lifespan. For example, quercetin glycosylated at position 3 or 3′, such as quercetin-3-O-glycoside and quercetin 3′-O-glycoside extended lifespan by 23% and 12% in *C. elegans* [34,35]. Therefore, it is speculated that glycosylated flavonoids identified in PE fruit polyphenols including quercetrin and spiraeoside may exert positive effect on lifespan. Moreover, some polyphenols with anti-aging ability were found to modulate the lifespan via insulin/insulin-like growth factor 1 signaling (IIS) pathway, closely connected with the metabolism, lifespan, and stress resistance of nematodes [33]. For instance, caffeic, dihydrocaffeic protocatechuic, and gallic acids executed their function via modulation of IIS pathway components, through promotion of daf-16, sod-3, and/or sir-2.1 gene expression [36,37]. This suggested that our further study can focus on the structure–activity relationship of PE fruit polyphenols on prolonging lifespan at the genomic levels.

#### 3.5.3. PE Fruit Polyphenols Inhibited the Cholinesterase Activities in *C. elegans*

**Figure 5:**
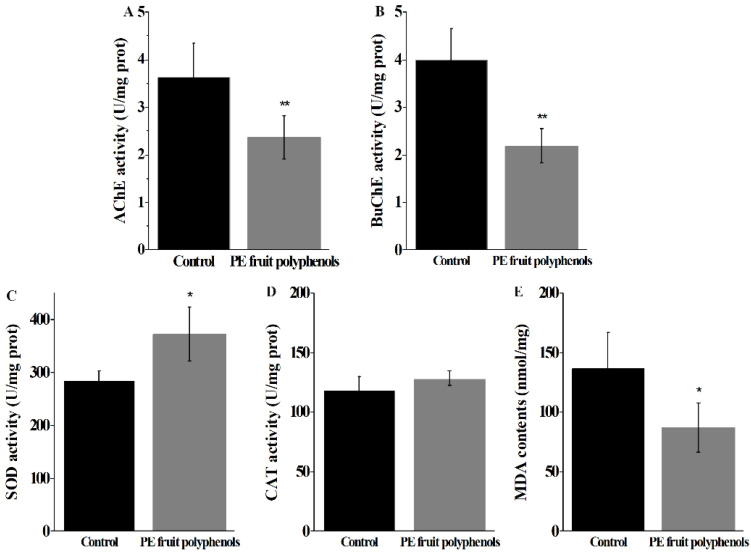
The effect of PE fruit polyphenols on the activity of AChE (**A**), BuChE (**B**), SOD (**C**), CAT (**D**) and the contents of MAD (**E**) in *C. elegans*. * *p* < 0.05 was significant from the control, and ** *p* < 0.01 was extremely significant from the control.

Cholinergic transmission is suggested to be involved in many age-related behaviors of *C. elegans*, including locomotion, egg laying, feeding, and mating. Among these, AChE and BuChE are responsible for regulating the quantity of the choline at neuronal junctions. As shown in Figure 5A,C, elegans supplemented with PE fruit polyphenols resulted in a decrease in AChE activity to a marked extent (34.71%) compared to the control groups (*p* < 0.01). Similarly, the activity of BuChE reduced with a significant inhibition rate of 45.38% in treated C. elegans (*p* < 0.01), which was better than that of AChE (Figure 5B). These results were consistent with the plummet in anti-cholinesterase activity observed in vitro (Section 3.4), verifying that PE fruit polyphenols had strong anti-cholinesterase activities.

Sustained oxidative damage accumulates with the increased age, accelerating the development of AD. It is known that inhibition of AChE and BuChE is a promising target for improvement in AD and aging process [28]. In the present study, PE fruit polyphenols was found to remarkably decline the activity of AChE and BuChE in vitro and in *C. elegans*. The effect of polyphenol monomers or polyphenol extract on cholinesterase has also been studied using other models in previous studies. It was reported recently by Tota et al. [38] that chronic 20 and 50 mg/kg of quercetin treatment for 21 days in adult male mice resulted in a significant reduction in AChE activity by 34.21% and 7.89%, respectively. Similarly, consumption of 2% w/v/day rosemary tea for 4 weeks decreased AChE activity in cerebral cortex (29.93%), midbrain (21.31%), cerebellum (31.30%), and striatum (24.56%) of mice brain, relatively. Further analysis revealed the presence of diterpenes, flavonoids, and hydroxycinnamic derivatives which were the major active compounds [39]. Besides, administration of 0.5% green tea extract for 8 weeks induced AChE activity decline from 278.2 ± 13.8 to 214.8 ± 41.7 nmoles/mg protein/min in the cerebrum of old rats [40]. In short, these findings verified that polyphenols exhibit dramatic inhibitory effect on cholinesterase activity, suggesting they are preferential for dietary interventions and nutritional food and effective in improving cognitive deficit and age-related diseases.

#### 3.5.4. PE Fruit Polyphenols Enhanced Antioxidant Enzymes Activities and Reduced MDA Level in *C. elegans*

As one of the most abundant antioxidant enzymes in organisms, SOD is the first line to maintain the balance of oxidation and antioxidation. It can reduce oxidative damage of cells by catalyzing •O_2_^−^ to O_2_ and H_2_O_2_ [32]. As shown in Figure 5C, SOD activity increased by 30.74% in polyphenols-treated *C. elegans* compared to control group (*p* < 0.05). CAT can rapidly decompose H_2_O_2_ into oxygen and water, and then react with peroxidase to form an antioxidant reaction chain [41]. It was found that PE fruit polyphenols induced the activity of CAT slightly increased by 8.42% in *C. elegans* (Figure 5D), whereas there was no significant difference compared with the control group (*p* > 0.05). Excessive oxidative damage not only damages cells, but also causes the occurrence of lipid peroxidation, ultimately forming the final product MDA [32]. In our data, the level of MDA in PE fruit polyphenol-treated group decreased by 36.25% compared with control exhibited in Figure 5E (*p* < 0.05). These results demonstrated that supplementation of PE fruit polyphenols could distinctly improve the antioxidant enzymes activity and reduce the MDA level in *C. elegans*.

The endogenous defense systems of organism mainly comprised of antioxidant enzymes, such as SOD, CAT, glutathione peroxidase, responsible for maintaining the oxidative equilibrium [6]. However, the endogenous defense systems are gradually reduced with increasing age. In recent years, dietary polyphenols have been gaining increasing scientific interest as important exogenous antioxidants. In our study, PE fruit polyphenols showed strong antioxidant activity with effectively enhancing the antioxidant defense systems, including improve enzymes activity (SOD and CAT) and reducing the MDA level, as well as scavenging DPPH, ABTS·^+^, OH· radicals and reducing ferric ion. Oxidative damage caused by excess free radicals and limited antioxidant defense system is widely regarded as a main account for aging process. Thus, it is convincing that PE fruit polyphenols might exert antioxidant ability to help resist the biological aging process of *C. elegans*, including enhancement in thermotolerance, extending the lifespan, and inhibition of cholinesterase (AChE and BuChE) activity.

## 4. Conclusions

In this study, RSM method was used to extract polyphenol from PE fruit, achieving a maximum TPC yield of 114.01 ± 2.31 mg GAE/g DW. PE fruit polyphenols was found to decelerate marks of aging in *C. elegans*, including enhancing the thermal resistance, prolonging the lifespan, and inhibition of AChE and BuChE activity. The improvement of aging process exerted by PE fruit polyphenols was suggested to be mediated through the marked antioxidant properties (including scavenging of free radicals, increase in antioxidant enzymes SOD and CAT, and decrease in MDA level). These activities might be interrelated with the presence of abundant flavonols and phenolic acids identified in our PE fruits, such as quercetin, myricetin, ellagic acid, gallic acid, chlorogenic acid, and their glycosides. Hence, PE fruit polyphenols have the potential to decelerate aging process and benefit human health, contributing to the future application in pharmaceutical, food, and cosmetic industry. However, the anti-aging properties of PE fruit polyphenols warrant further studies with larger groups of animals, on their individual active constituents and action mechanism.

## Figures and Tables

**Table 1:** Coded values for BBD and experimentally observed responses.

Run	A: Ethanol Concentration (%)	B: Liquid-Solid Ratio (mL/g)	C: Extraction Temperature (°C)	Y: TPC Yield (mg GAE/g DW)
1	60	20	40	93.20
2	45	20	50	101.10
3	60	20	40	93.20
4	75	15	40	88.03
5	60	20	40	90.88
6	75	25	40	89.77
7	60	25	30	103.14
8	75	20	30	86.69
9	60	15	30	95.00
10	60	20	40	91.81
11	45	20	30	99.71
12	60	20	40	92.27
13	60	15	50	93.03
14	75	20	50	88.09
15	45	15	40	91.69
16	45	25	40	114.35
17	60	25	50	100.81

**Table 2:** Phenolic compounds tentatively identified in PE fruit via UPLC-ESI-QTOF-MS in positive and negative ionization modes.

No	Rt (min)	(m/z) [M-H]^−/+^	Tentative Identification	Proposed Formula	Molecular Weight
Hydroxybenzoic acids					
1	2.415	153.0193 [M + H]^−^	Protocatechuic acid	C_7_H_6_O_4_	154.0266
2	2.337	169.0139 [M + H]^−^ 171.0292 [M + H]^+^	Gallic acid	C_7_H_6_O_5_	170.0215
3	3.642	183.0288 [M + H]^−^	3-O-Methylgallate	C_8_H_7_O_5_^−^	184.0372
4	5.232	197.0440 [M + H]^−^ 199.0590 [M + H]^+^	Syringic acid	C_9_H_10_O_5_	198.0528
5	2.533	243.0492 [M + H]^−^	1-O-Galloylglycerol	C_10_H_12_O_7_	244.0583
6	2.990	185.0441 [M + H]^+^	4-O-Methylgallic acid	C_8_H_8_O_5_	184.0372
7	4.658	300.9981 [M + H]^−^ 303.0145 [M + H]^+^	Ellagic acid	C_14_H_6_O_8_	302.0063
8	1.527	331.0653 [M + H]^−^ 333.0799 [M + H]^+^	beta-Glucogallin	C_13_H_16_O_10_	332.0743
9	0.821	361.0410 [M-H]^−^ 363.0567 [M + H]^+^	2-O-Galloylgalactaric acid	C_13_H_14_O_12_	362.0485
10	2.342	375.0575 [M-H]^−^ 399.0526 [M + Na]^+^	1-Methyl 2-galloylgalactarate	C_14_H_16_O_12_	376.0642
11	2.839	379.0087 [M + Cl]^−^ 345.0426 [M + H]^+^	2-O-Galloyl-1,4-galactarolactone	C_13_H_12_O_11_	344.038
12	2.525	483.0736 [M-H]^−^	2,6-Digalloylglucose	C_20_H_20_O_14_	484.0853
13	3.429	483.0780 [M-H]^−^ 507.0729 [M + Na]^+^	1-O,6-O-Digalloyl-beta-D-glucose	C_20_H_20_O_14_	484.0853
14	2.034	495.0754 [M-H]^−^ 497.0912 [M + H]^+^	3,4-Di-O-galloylquinic acid	C_21_H_20_O_14_	496.0853
15	2.023	357.0462 [M + H]^+^	Chebulic acid	C_14_H_12_O_11_	356.0380
Hydroxycinnamic acids					
16	4.557	177.0190 [M + H]^−^	Esculetin	C_9_H_6_O_4_	178.0266
17	3.526	179.0349 [M + H]^−^	Caffeic acid	C_9_H_8_O_4_	180.0423
18	2.439	311.0396 [M + H]^−^ 313.0557 [M + H]^+^	Caftaric acid	C_13_H_12_O_9_	312.0481
19	2.651	369.0436 [M-H]^−^ 371.0620 [M + H]^+^	2-O-Caffeoylhydroxycitric acid	C_15_H_14_O_11_	370.0536
20	2.881	369.0790 [M-H]^−^	Fraxin	C_16_H_18_O_10_	370.0900
21	3.326	383.0608 [M-H]^−^ 385.0747 [M + H]^+^	2-O-Feruloylhydroxycitric acid	C_16_H_16_O_11_	384.0693
22	1.759	391.0475 [M + Cl]^−^	Caffeic acid 3-O-glucuronide	C_15_H_16_O_10_	356.0743
23	1.800	297.0598 [M + H]^+^	Caffeoylmalic acid	C_13_H_12_O_8_	296.0532
24	2.660	355.1001 [M + H]^+^ 377.0816 [M + Na]^+^	Chlorogenic acid	C_16_H_18_O_9_	354.0951
25	4.796	373.0750 [M + H]^+^	2-O-Caffeoylglucarate	C_15_H_16_O_11_	372.0693
Flavanones					
26	6.525	271.0594 [M + H]^−^ 273.0743 [M-H]^+^	Naringenin	C_15_H_12_O_5_	272.0685
27	4.840	427.1794 [M-H]^−^	Heteroflavanone B	C_24_H_28_O_7_	428.1835
28	6.519 6.525	433.1108 [M-H]^−^ 435.1303 [M + H]^+^	Naringenin-7-O-glucoside	C_21_H_22_O_10_	434.1213
29	8.565	579.1503 [M-H]^−^	6′′-p-Coumaroylprunin	C_30_H_28_O_12_	580.1581
30	11.954	405.1541 [M + H]^+^	Citromitin	C_21_H_24_O_8_	404.1471
31	7.710	417.1533 [M + H]^+^	4′-Methylliquiritigenin 7-rhamnoside	C_22_H_24_O_8_	416.1471
32	5.803	465.1341 [M + H]^+^	Hesperetin 5-O-glucoside	C_22_H_24_O_11_	464.1319
33	3.167	689.1165 [M + Cl]^−^	Hesperetin 5,7-O-diglucuronide	C_28_H_30_O_18_	654.1432
Flavan-3-ols					
34	4.909	481.0958 [M-H]^−^	(-)-Epigallocatechin 3′-glucuronide	C_21_H_22_O_13_	482.106
35	2.798	323.0729 [M + H]^+^	Leucodelphidin	C_15_H_14_O_8_	322.0689
36	1.105	867.1327 [M-H]^−^	Theaflavin 3,3′-digallate	C_43_H_32_O_20_	868.1487
37	2.553	621.0720 [M + H]^+^	Tannin	C_26_H_20_O_18_	620.0650
Flavonols					
38	2.627	411.0528 [M + Cl]^−^	Limocitrol	C_18_H_16_O_9_	376.0794
39	7.041	287.0553 [M-H]^+^	Fisetin	C_15_H_10_O_6_·xH_2_O	286.0477
40	7.042	431.0979 [M-H]^−^	Afzelin	C_21_H_20_O_10_	432.1056
41	6.316	447.0897 [M-H]^−^ 449.1091 [M + H]^+^	Quercitrin	C_21_H_20_O_11_	448.1006
42	6.316	303.0501 [M + H]^+^	Quercetin	C_15_H_10_O_7_	302.0427
43	7.906	461.1060 [M-H]^−^	Kaempferide 7-glucoside	C_22_H_22_O_11_	462.1162
44	5.463	463.0863 [M-H]^−^	Isoquercetin	C_21_H_20_O_12_	464.0955
45	5.631	463.0863 [M-H]^−^	Spiraeoside	C_21_H_20_O_12_	464.0955
46	6.211	469.0483 [M + Cl]^−^	Quercetin 7-xyloside	C_20_H_18_O_11_	434.0849
47	5.458	319.0435 [M + H]^+^	Myricetin	C15H10O8	318.0376
48	4.727	341.0328 [M + Na]^+^	Gossypetin	C_15_H_10_O_8_	318.0376
49	4.796	507.1093 [M-H]^−^	Syringetin-3-O-galactoside	C_23_H_24_O_13_	508.1217
50	2.674	529.0789 [M + Cl]^−^	Laricitrin 3-glucoside	C_22_H_22_O_13_	494.106
51	2.714	609.1281 [M-H]^−^	6′′-O-Caffeoylastragalin	C_30_H_26_O_14_	610.1323
52	5.581	625.1426 [M-H]^−^	Quercetin 4′,7-diglucoside	C_27_H_30_O_17_	626.1483
53	3.301	419.0990 [M + H]^+^	Kaempferol 3-alpha-L-arabinopyranoside	C_20_H_18_O_10_	418.0900
54	4.183	667.0737 [M + Cl]^−^	Myricetin 7-(6′′-galloylglucoside)	C_28_H_24_O_17_	632.1013
55	2.541	675.1030 [M + Cl]^−^	Nelumboside	C_27_H_28_O_18_	640.1276
56	4.727	434.9980 [M + Na]^+^	Quercetagetin 3-methyl ether 7-O-sulfate	C_16_H_12_O_11_S	412.0100
57	0.910	723.2186 [M-H]^−^	Natsudaidain 3-(4-O-3-hydroxy-3-methylglutaroylglucoside)	C_33_H_40_O_18_	724.2215
58	5.232	451.0887 [M + H]^+^	Myricetin 3-xyloside	C_20_H_18_O_12_	450.0798
59	6.525	463.0887 [M + H]^+^	Kaempferol 3-glucuronide	C_21_H_18_O_12_	462.0798
60	5.794	473.0717 [M + Na]^+^	Myricetin 3-arabinoside	C_20_H_18_O_12_	450.0798
61	1.827	837.1519 [M + Cl]^−^	Quercetin 7-glucuronoside 3-sophoroside	C_33_H_38_O_23_	802.1804
62	2.039	479.0858 [M + H]^+^	Quercetin 3-O-glucuronide	C_21_H_18_O_13_	478.0747
63	5.582	481.0944 [M + H]^+^	Myricetin 3-glucoside	C_21_H_20_O_13_	480.0904
64	5.458	487.0868 [M + Na]^+^	Myricitrin	C_21_H_20_O_12_	464.0955
65	4.141	495.0767 [M + H]^+^	Myricetin 3-glucuronide	C_21_H_18_O_14_	494.0697
66	3.660	507.1097 [M + H]^+^	Quercetin 3-O-(6′′-acetyl-glucoside)	C_23_H_22_O_13_	506.1060
Anthocyanidins					
67	3.302	417.0806 [M-H]^−^	Cyanidin 3-arabinoside	C_20_H_19_O_10_	419.0978
68	5.463	451.0853 [M + Cl]^−^	Pelargonidin 3-rhamnoside	C_21_H_21_O_9_^+^	417.1186
69	0.672	603.1002 [M-H]^−^	Pelargonidin 3-O-3′′,6′′-O-dimalonylglucoside	C_27_H_25_O_16_^+^	604.1064
70	3.183	645.1292 [M + Cl]^−^	Cyanidin 3-galactoside-5-glucoside	C_27_H_31_O_16_^+^	610.1534
71	1.113	661.1228 [M + Cl]^−^	Delphinidin 3-sophoroside	C_27_H_31_O_17_	627.1561
72	12.700	610.1867 [M + H]^+^	Peonidin 3-rhamnoside 5-glucoside	C_28_H_33_O_15_	609.1819
Dihydroflavonols					
73	5.689	449.1048 [M-H]^−^	Neoastilbin	C_21_H_22_O_11_	450.1162
Flavones					
74	6.519	461.0708 [M-H]^−^	Scutellarein 5-glucuronide	C_21_H_18_O_12_	462.0798
75	7.899	489.1036 [M-H]^−^	2′′-O-Acetylisoorientin	C_23_H_22_O_12_	490.1111
76	2.541	499.0691 [M + Cl]^−^	2′-Hydroxyisoorientin	C_21_H_20_O_12_	464.0955
77	0.752	409.0930 [M + Na]^+^	Chrysin 5-xyloside	C_20_H_18_O_8_	386.1002
78	1.559	685.1240 [M + Cl]^−^	6′′-Malonylapiin	C_29_H_30_O_17_	650.1483
Isoflavones					
79	4.636	465.0620 [M + Cl]^−^	Daidzein 4′-O-glucuronide	C_21_H_18_O_10_	430.0900
80	6.382	465.0974 [M + Cl]^−^	Ononin	C_22_H_22_O_9_	430.1264
81	0.657	517.1393 [M-H]^−^	Medicarpin 3-O-(6′-malonylglucoside)	C_25_H_26_O_12_	518.1424
82	4.846	405.1706 [M + H]^+^	Osajin	C_25_H_24_O_5_	404.1624
83	5.223	419.0613 [M + H]^+^	Shoyuflavone C	C_19_H_14_O_11_	418.0536
84	7.041	455.0916 [M + Na]^+^	Genistin	C_21_H_20_O_10_	432.1056

## Data Availability

The authors declare that all data supporting the findings of this study are available within the paper. Raw data that support the findings of this study are available from the corresponding author upon reasonable request.

## Supplementary Materials

The following are available online at https://www.mdpi.com/article/10.3390/nu14040857/s1. Table S1. Analysis and statistical parameters of regression model.
