# Supplementary material for: The Composition and Anti-Aging Activities of Polyphenol Extract from Phyllanthus emblica L. Fruit
Source: Nutrients. 2022 Feb 18;14(4):857. doi: 10.3390/nu14040857 (PMC8878974; doi:10.3390/nu14040857)
Supplement: Supplementary file 1 [file nutrients-14-00857-s001.zip › nutrients-1577810-supplementary.pdf]

Table S1. Analysis and statistical parameters of regression model.

**Source**	**Sum of Squares**	**Df**	**Mean Square**	**F–Value**	**p–value**	significant
Model	762.44	9	84.72	30.02	< 0.0001	**
A–Ethanol concentration	368.15	1	368.15	130.44	< 0.0001	**
B–Liquid-solid ratio	203.21	1	203.21	72.00	< 0.0001	**
C–Extraction temperature	0.29	1	0.29	0.10	0.7599	
AB	109.41	1	109.41	38.77	0.0004	**
AC	2.5E–005	1	2.5E–005	8.858E–006	0.9977	
BC	0.032	1	0.032	0.011	0.9177	
A^2	0.18	1	0.18	0.063	0.8097	
B^2	63.80	1	63.80	22.61	0.0021	**
C^2	14.10	1	14.10	5.00	0.0605	
Residual	19.76	7	2.82			
Lack of Fit	15.88	3	5.29	5.47	0.0672	Not significant
Pure Error	3.87	4	0.97			
Cor Total	782.20	16				

Note: * indicates the significant difference ($p < 0.05$), and ** indicates the highly significant difference ($p < 0.01$).
